# Quantification of myocardial iron and fat—an experimental study with photon-counting detector CT

**DOI:** 10.1093/bjr/tqag020

**Published:** 2026-01-25

**Authors:** Philipp N Maintz, Tristan T Demmert, Thomas Flohr, Konstantin Klambauer, Lukas J Moser, Victor Mergen, Matthias Eberhard, Johannes M Froehlich, Hatem Alkadhi

**Affiliations:** Diagnostic and Interventional Radiology, University Hospital Zurich, University of Zurich, 8091 Zurich, Switzerland; Faculty of Medicine, Sigmund Freud University, 1020 Vienna, Austria; Diagnostic and Interventional Radiology, University Hospital Zurich, University of Zurich, 8091 Zurich, Switzerland; Diagnostic and Interventional Radiology, University Hospital Zurich, University of Zurich, 8091 Zurich, Switzerland; Department of Radiology and Nuclear Medicine, Maastricht University Medical Centre+, 6229 HX Maastricht, the Netherlands; Diagnostic and Interventional Radiology, University Hospital Zurich, University of Zurich, 8091 Zurich, Switzerland; Diagnostic and Interventional Radiology, University Hospital Zurich, University of Zurich, 8091 Zurich, Switzerland; Diagnostic and Interventional Radiology, University Hospital Zurich, University of Zurich, 8091 Zurich, Switzerland; Diagnostic and Interventional Radiology, University Hospital Zurich, University of Zurich, 8091 Zurich, Switzerland; KlusLab Research, 8032 Zurich, Switzerland; Diagnostic and Interventional Radiology, University Hospital Zurich, University of Zurich, 8091 Zurich, Switzerland

**Keywords:** tomography, X-ray computed, iron, haemochromatosis, beta-thalassemia, iron overload, myocardium

## Abstract

**Objectives:**

To determine the feasibility and accuracy of photon-counting detector (PCD)-CT for iron and fat quantification in the myocardium.

**Methods:**

Cylindrical tubes were filled with porcine myocardium and iron citrate with iron concentrations of 0-20 mg Fe g^−1^. Dilution series were prepared with myocardium and iron (*no-fat probes*) and with 5% fat (*fat probes*). The tubes were positioned in a chest phantom and were scanned with a calcium-scoring protocol on a PCD-CT. A re-parameterized 3-material decomposition was used to separate iron and fat from myocardium.

**Results:**

On virtual monoenergetic images, attenuation increased linearly with iron concentrations in both fat and no-fat probes. In no-fat probes, linear regression yielded a slope of 1.2 HU (mg Fe g^−1^)^−1^ with an intercept of 35.8 HU (*R*^2^ = 0.964). In the fat probes, the slope was similar at 1.1 HU (mg Fe g^−1^)^−1^, while the regression line shifted downwards by 6.1 HU with an intercept of 29.6 HU (*R*^2^ = 0.985). Iron maps separated fat from iron with calculated median fat fractions of 4.85 in the fat and 0.90 in the no-fat probes. In iron images, attenuation increased linearly with increasing iron concentrations, with similar slopes between fat and no-fat probes and negligible differences in the intercept.

**Conclusions:**

Experimental evidence indicates the feasibility and accuracy of PCD-CT for iron and fat quantification in the myocardium. Iron-specific 3-material decomposition eliminates the confounding effect of fat on myocardial iron quantification.

**Advances in knowledge:**

This study highlights the value of dual-energy CT with 3-material decomposition for quantifying iron and fat in the myocardium. Thus, CT could serve as alternative for the current reference standard MRI.

## Introduction

Iron overload cardiomyopathy results from excessive myocardial iron deposition, most commonly due to hereditary disorders of iron metabolism, haemoglobinopathy, or repeated blood transfusions.^1^ It is frequently encountered in patients with thalassemia major and hereditary haemochromatosis, where myocardial iron burden contributes substantially to morbidity and mortality.^2^

Cardiac MRI with T2*-weighted gradient echo sequences is the non-invasive reference standard for quantifying myocardial iron. This technique exploits the paramagnetic properties of iron, which shorten T2 and T2* relaxation times, and has established MRI as the widely adopted clinical standard.^3–5^ Despite its advantages, MRI has known limitations, including long examination times, contraindications in certain patients, and high costs. These challenges have prompted the search for complementary approaches that can provide comparable precision with greater practicality.^6^

Iron is inherently radiopaque due to its relatively high atomic number (*Z* = 26) compared to other body materials, which makes it potentially suitable for a CT-based detection and quantification. Preliminary experimental studies reported the feasibility of myocardial iron detection by CT but were limited by base material not using myocardium^7^ and did not account for potential confounding by fat.^7^^,^^8^ Fat shows divergent attenuation characteristics as compared to iron in single-energy CT, which can be overcome through 3-material decomposition using dual-energy CT, as previously shown in studies of the liver.^9–11^

The purpose of this experimental study was to determine the feasibility and accuracy of photon-counting detector (PCD)-CT for myocardial iron and fat quantification, using both virtual monoenergetic images (VMIs) and iron maps computed with a 3-material decomposition algorithm.

## Methods

### Phantom

Fresh porcine myocardium was obtained from a local butcher immediately post-mortem. Excess epicardial fat was carefully trimmed to achieve consistent tissue composition. The myocardium was then minced twice using a commercial meat grinder, followed by further homogenization using a handheld immersion blender (Menagros AG, Switzerland) to achieve a uniform paste-like consistency with as little air as possible.

Two series of probes were prepared in a standardized manner. In the first series, only myocardium and iron were combined, aiming to replicate physiologic and clinically relevant pathologic myocardial iron concentrations. Iron was added in the form of iron (III) citrate monohydrate powder (C_6_H_5_FeO_7_·H_2_O, molar mass 262.96 g mol^−1^; Sigma-Aldrich, Merck Switzerland, Buchs, Switzerland). The target iron concentrations were 0, 1, 2, 3, 4, 5, 7.5, 10, 15, and 20 mg Fe g^−1^ dry weight equivalent to 0, 0.2, 0.4, 0.6, 0.8, 1.0, 1.5, 2.0, 3.0, and 4.0 mg Fe mL^−1^ wet weight, respectively (Table 1), covering both the physiological range and levels observed in pathology.^3^^,^^12^ Wet-weight iron concentrations used for the phantoms were calculated based on a wet-to-dry ratio of 5, as previously described.^3^ The calculated wet-iron concentrations were used to prepare the phantoms. A total of 45 g of myocardial paste was combined with the respective iron amount. The final iron concentrations and gravimetric accuracy of iron addition were documented, with deviations from target values below 1.2%, confirming precise phantom preparation. These probes, composed of iron and myocardium only, were designated as *no-fat probes*.

**Table 1.** Phantom iron concentrations and corresponding CT attenuation values from 3-material decomposition iron maps in the fat and the no-fat probes.

Wet-weight iron concentration (mg mL^−1^)	Dry-weight equivalent (mg Fe g^−1^)	CT attenuation for iron with 5% fat (HU)	CT attenuation for iron without fat (HU)	Measured fat fraction in probes with 5% fat (%)	Measured fat fraction in probes without fat (%)
0.0	0.0	−5.8 ± 5.0	−4.7 ± 5.0	2.2 ± 3.9	0.8 ± 6.4
0.2	1.0	−1.7 ± 4.5	4.3 ± 3.2	3.8 ± 4.2	0.6 ± 5.5
0.4	2.0	1.6 ± 3.6	−3.7 ± 8.1	5.5 ± 3.6	−1.6 ± 7.1
0.6	3.0	2.2 ± 1.0	3.7 ± 4.1	4.8 ± 4.7	3.0 ± 7.1
0.8	4.0	2.6 ± 3.6	0.1 ± 11.3	3.1 ± 5.0	3.0 ± 10.0
1.0^a^	5.0	4.3 ± 2.7	3.7 ± 5.3	5.0 ± 3.0	2.5 ± 5.9
1.5	7.5	3.2 ± 1.1	7.8 ± 4.3	6.4 ± 1.1	−0.8 ± 6.4
2.0	10.0	15 ± 6.3	12.4 ± 3.1	4.9 ± 7.7	1.0 ± 3.6
3.0	15.0	17.6 ± 2.4	19.5 ± 4.2	4.7 ± 3.7	0.1 ± 6.7
4.0	20.0	31.4 ± 2.1	27.7 ± 5.4	7.5 ± 2.6	2.8 ± 6.8

Data represent mean values across all 7 regions of interest for each probe at different iron concentrations.

a The 1 mg Fe g^−1^ measurement deviated substantially from the linear trend and, upon quality-control review, was attributed to an error during probe preparation and excluded from the analysis.

In the second series, myocardium was mixed with 5% porcine fat reflecting stereological estimates of myocardial fat content^13^^,^^14^ and were designated as *fat probes*. In the probes containing myocardium, fat, and iron, 42.75 g of myocardial paste was mixed with 2.25 g of fat and the respective iron amount. All mixtures were homogenized using an impeller stirrer (Unguator Pro, Gako GmbH, Schesslitz, Germany) at 2150 rpm for 1 min and 40 s, carefully maintaining the temperature below 35 °C, monitored using a digital infrared thermometer (Stanley Black & Decker, New Britain, CT, United States). Subsequently, the probes were transferred into cylindrical polypropylene tubes (50 mL Aponorm screw-lid jar; WEPA GmbH & Co. KG, Hillscheid, Germany) to create the final phantoms. These were sealed airtight and stored at 2 °C to minimize oxidative degradation prior to imaging. In addition, a probe containing pure myocardium and a probe containing pure fat were prepared for calibration purposes.

For the CT scans, probes were placed in groups of 3 in the central 15 cm diameter bore of a commercial anthropomorphic chest phantom (QRM, Möhrendorf, Germany) to account for physiological human attenuation values.

### CT data acquisition and data reconstruction

All probes were scanned with a first generation dual-source PCD-CT system (NAEOTOM Alpha, Siemens Healthineers AG, Forchheim, Germany) using our routine institutional protocol for cardiac calcium scoring. Scans were acquired in a prospectively electrocardiography (ECG)-triggered spectral mode (QuantumPlus, Siemens) with a simulated electrocardiogram with 60 beats per minute. Tube voltage was set to 120 kVp, image quality level to 20, and the detector collimation was 144 × 0.4 mm. The average CTDI_vol_ was 2.2 ± 0.16 mGy. Scans were reconstructed as VMIs at 70 keV and as spectral post-processing (SPP) images representing dual-energy data, with a slice thickness 2 mm, increment of 1.5 mm, and using a quantitative soft tissue kernel (Qr40) with a 512 × 512 matrix.

### Data post-processing and image analysis

A commercial 3-material decomposition algorithm (liver virtual non-contrast, syngo Dual Energy, Siemens) was used to develop the algorithm for differentiating fat, myocardium, and iron. This 3-material decomposition from dual-energy data is possible under certain assumptions that reduce the task to a modified 2-material decomposition.^15^ One base material is a mixture of soft tissue and fat. Volume conservation is assumed—each voxel can contain either 100% soft tissue, 100% fat, or a mixture of both materials (eg, 60% soft tissue, 40% fat). The second base material is a material with a high atomic number (*Z*) and a negligible volume fraction. By material decomposition, images can be calculated with or without the high-*Z* material. In the application, liver parenchyma as the soft tissue and iodine as the high-*Z* material are parametrized by default.^15^ These default settings can be modified to designate myocardium as the reference soft tissue and iron as the high-*Z* material. Therefore, the attenuation values of pure myocardium and pure fat must be determined at both low and high threshold energies, and the dual-energy ratio (DER) of iodine must be replaced by the DER of iron.

**Figure 1.**
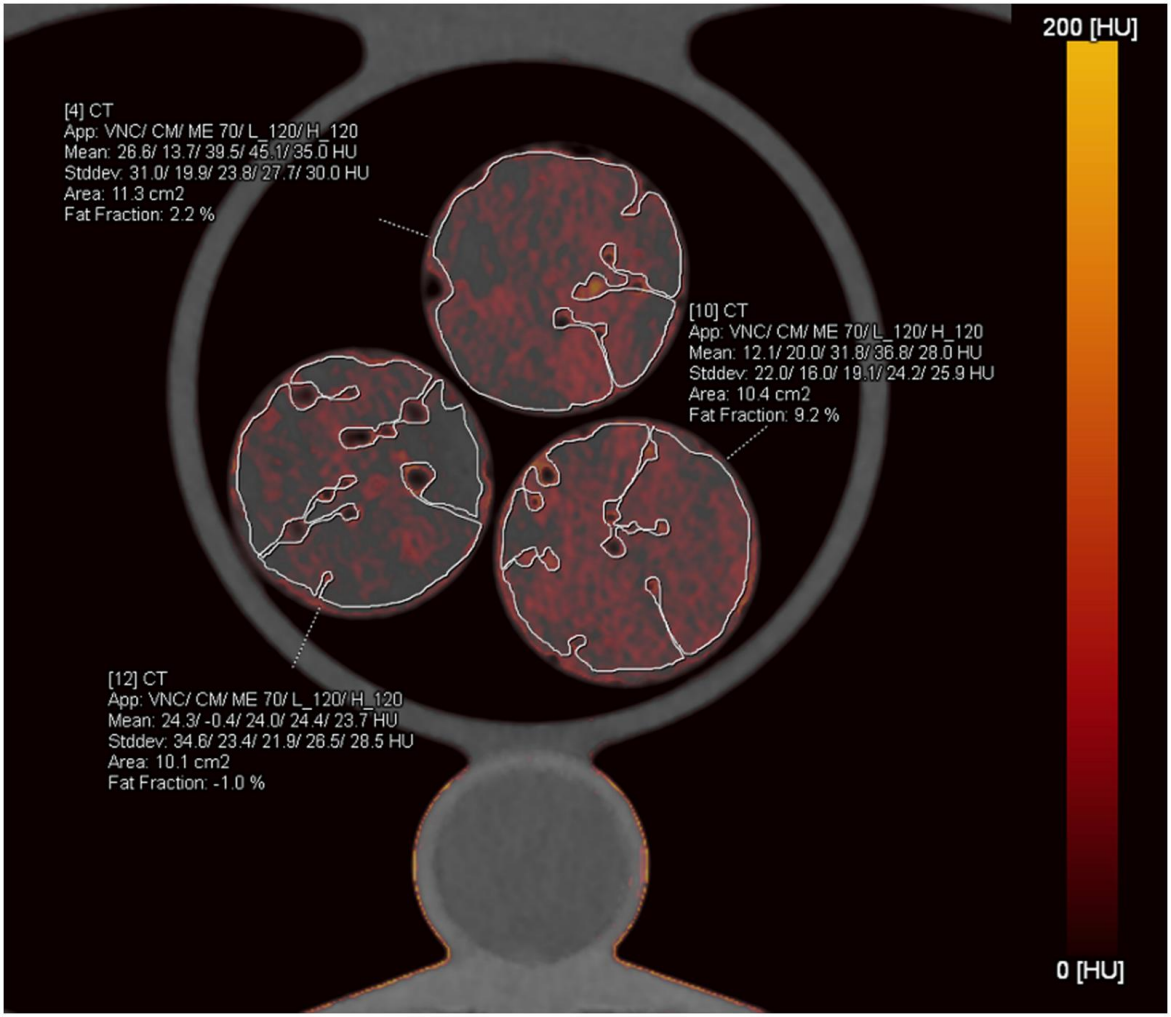
Image illustrating the free-hand region-of-interest placement in the probes evaluated with the dual-energy-based 3-material decomposition. The contrast material (CM) for this modified application was iron. The 3-material decomposition algorithm yielded iron values in these regions of interest (ROIs) of 13.7 HU for the probe in the top, 20 HU for the one on the left and −0.4 HU for the one on the right. The fat fraction in percent (%) was obtained from each ROI.

For calibration, the tubes containing pure myocardium and pure fat were scanned and the mean attenuation values in the low and high threshold images were measured in 15 regions-of-interest (ROIs) each. We used a DER of 1.7 for iron, being adapted from the literature for PCD-CT scans with 120 kVp.^16^ Finally, this 3-material decomposition algorithm provides iron maps. Measurements were performed in random order by placing 7 freehand ROIs in different slices to ensure comprehensive coverage of the entire long axis of the probes. The freehand ROIs were placed to avoid air bubbles and edges (Figure 1). Iron-specific attenuation values (in HU) and fat fractions (in percent, %) were obtained from each ROI.

On VMI, attenuation measurements (in HU) were performed by placing 15 circular ROIs in air-free homogenous parts of the probes, hereby eliminating deviations from air bubbles trapped inside the material. The ROIs were placed over the full height of the containers in all probes.

### Statistical analysis

Means and SDs are reported for normally distributed parameters, medians, and interquartile ranges (IQRs) for non-normally distributed parameters. For VMI and iron maps, attenuation measurements were modelled as a function of the known iron concentrations using linear regression, reporting slope, intercept, and *R*^2^. At each iron concentration, the iron-specific attenuation values from the fat probes were compared with those from the no-fat probes using the Wilcoxon rank-sum (Mann–Whitney *U*) test. Calculated fat concentrations from 3-material decomposition were compared with the known fat concentrations in the probes. Comparisons were performed on probe-level values by averaging multiple ROIs per probe. *P*-values were adjusted across concentrations with the Holm method. Data analysis and visualization were performed using Microsoft Excel All analyses were conducted using Microsoft Excel (Microsoft Corporation, Redmond, WA, United States) and R (version 2025.05, R Foundation for Statistical Computing, Vienna, Austria).

## Results

### Evaluation of VMIs

**Figure 2.**
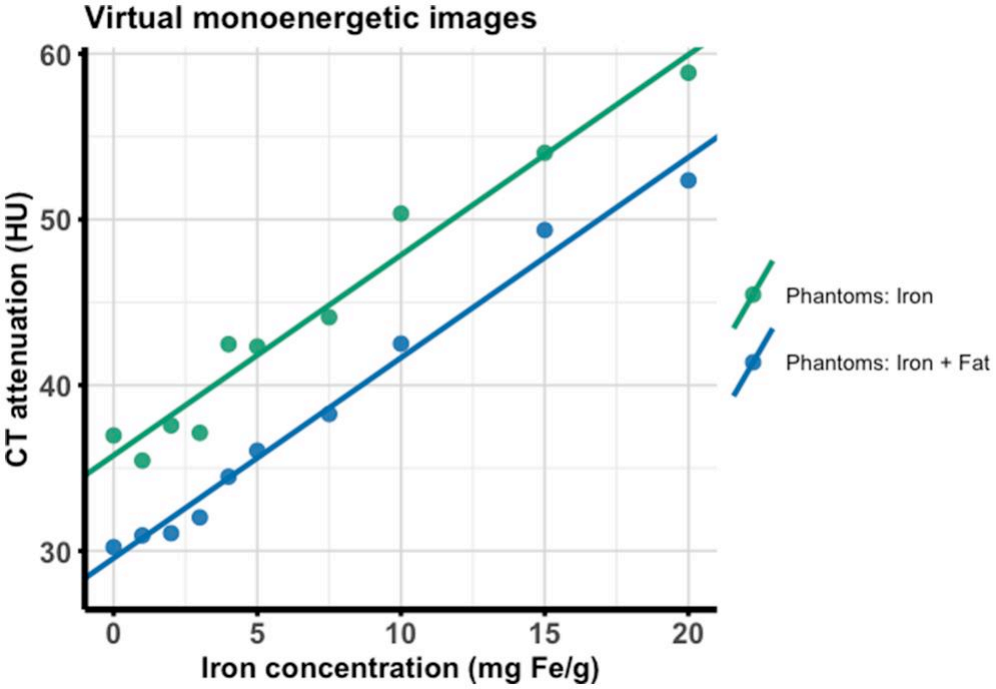
CT attenuation (in HU) on virtual monoenergetic images at 70 keV as a function of iron concentration (in mg Fe g^−1^). Note the similar slope of the regression lines with an offset introduced by 5% myocardial fat.

On VMIs at 70 keV, attenuation increased linearly with higher iron concentrations from 0 to 20 mg Fe g^−1^ with identical slopes of 1.21 HU (mg Fe g^−1^)^−1^ in the fat and no-fat probes (Figure 2, Table 2). Intercepts with the *y*-axis were lower in the presence of fat, yielding a linear regression model of *y* = 1.21 HU (mg Fe g^−1^)× + 29.6 HU, *R*^2^=0.985, as compared to the no-fat probes, which yielded a model of *y* = 1.21 HU (mg Fe g^−1^)^−1^× + 35.8 HU, *R*^2^=0.964. Thus, the 2 regression lines were parallel, separated by a constant offset attributable to fat (see Figure 2). Consequently, accurate quantification of the iron content using VMI (comparable to a conventional single-energy CT scan) is not possible without knowledge of the fat content.

**Table 2.** Phantom iron concentrations and virtual monoenergetic images in the fat and the no-fat probes.

**Wet-weight iron concentration (mg mL** ^−1^ **)**	VMI CT-attenuation in fat probes (HU)	VMI CT-attenuation in no-fat probes (HU)
0.0	30.2 ± 8.2	37.0 ± 5.6
0.2	30.9 ± 6.9	35.5 ± 4.0
0.4	31.0 ± 7.7	37.6 ± 4.6
0.6	32.0 ± 8.2	37.1 ± 5.0
0.8	34.5 ± 7.4	42.5 ± 5.4
1.0	36.0 ± 7.9	42.3 ± 4.9
1.5	38.3 ± 7.6	44.1 ± 4.8
2.0	42.5 ± 8.0	50.4 ± 7.3
3.0	49.4 ± 10.0	54.0 ± 7.3
4.0	52.4 ± 7.7	58.9 ± 9.4

Data represent mean values across all 15 regions of interest for each probe at different iron concentrations.

Abbreviation: VMI=virtual monoenergetic image.

### Evaluation of iron maps

**Figure 3.**
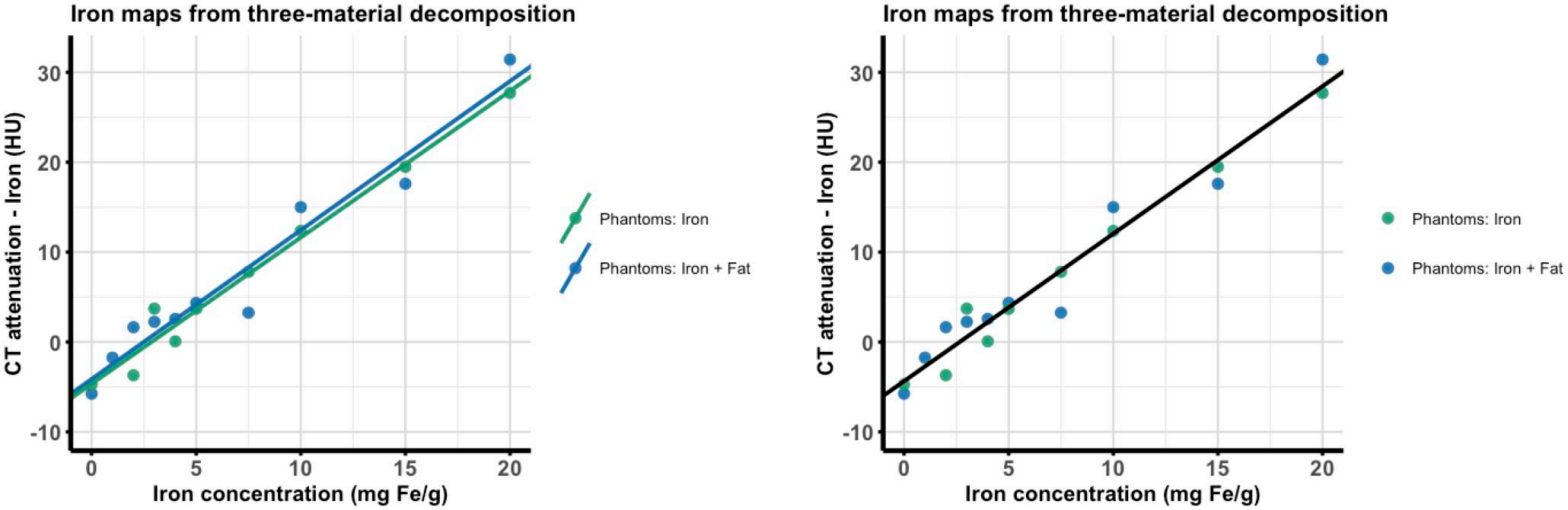
CT attenuation (in HU) on iron maps from 3-material decomposition using dual-energy data as a function of iron concentration (in mg Fe g^−1^). (Left) After the separation of fat from iron, regression lines were similar for both the probes with and without myocardial fat. (Right) Pooled fit for fat independent iron quantification on iron maps from 3-material decomposition using dual-energy data.

On iron maps, a common linear relationship between attenuation and the known iron concentration was observed for both phantom series with and without fat (Figure 3A). The 1 mg Fe g^−1^ measurement deviated substantially from the linear trend and, upon quality-control review, was attributed to an error during probe preparation and was therefore excluded from the further analysis.

Linear regression models yielded *y* = 1.66 HU (mg Fe g^−1^)^−1^·×−4.13 HU (*R*^2^=0.95) for the fat probes and *y* = 1.63 HU (mg Fe g^−1^)^−1^· × −4.66 HU (*R*^2^=0.98) for the no-fat probes, indicating linearity over the entire range of iron concentrations (0-20 mg g^−1^). The difference in the *y*-axis intercepts was small (Δ*y* = 0.53 HU), consistent with effective removal of fat through 3-material decomposition. Across all iron levels (0-20 mg Fe g^−1^, excluding 1 mg Fe/g), Wilcoxon rank-sum test showed no significant differences between measurements with and without fat (*P* < .05, for all), and a Wilcoxon signed-rank test on concentration-wise mean differences detected no systematic offset (median difference ≈0 HU; *P* > .9). Because the regression lines were essentially superimposable, both series were summarized by a single, fat-independent calibration and plotted in one panel (pooled fit: *y* = 1.64 HU (mg Fe g^−1^)^−1^· × −4.36 HU; *R*^2^=0.959) (Figure 3B).

The iron-specific 3-material decomposition yielded a median fat fraction of 4.85 (IQR: 4.0; 5.4) in the fat probes (nominal fat fraction 5%) and 0.90 (IQR: 0.2; 2.7) in the no-fat probes (see Table 1).

## Discussion

This experimental study demonstrates the feasibility of myocardial iron quantification at clinically relevant concentrations with PCD-CT, using a routine spectral protocol typically used for coronary calcium scoring. We found a linear correlation between CT attenuation in the myocardium and iron concentration in both VMI and iron maps calculated with an iron-specific 3-material decomposition algorithm. Fat confounds on iron measurements as it consistently lowers the CT attenuation in the myocardium. Spectral 3-material decomposition effectively separated fat from myocardial tissue, allowing accurate iron quantification even in the presence of fat.

Previous studies have indicated the added value of dual-energy CT for liver iron quantification. Fischer et al.^11^ showed in an *ex vivo* study that virtual non-iron images, generated via an iron-specific 3-material decomposition algorithm, allow accurate liver fat quantification independent of iron. These findings were subsequently confirmed by Ma et al.^10^ in a rat model and in a phantom study by Du et al.,^17^ who both showed that 3-material decomposition preserves accurate fat quantification despite co-existing iron. A recent study indicated also the feasibility of dual-energy based spectral localizer radiographs for quantifying both liver iron and fat. This approach highlights the utility of dual-energy CT in complex tissue environments comprising multiple materials. Hollý et al.^18^ used a PCD-CT scanner applying a 3-material algorithm for liver fat quantification in the presence of iron and found promising results, demonstrating that spectral PCD-CT can reliably disentangle overlapping material signals even in challenging mixed-tissue environments. Building on this work, we adapted the 3-material decomposition algorithm to myocardial tissue, using myocardium-specific attenuation profiles and tailored calibration, thereby transferring the concept into a new and potentially clinically relevant domain. Leveraging the inherent spectral capabilities of PCD-CT, the routine low-dose, non-contrast calcium scoring protocol enables myocardial iron quantification without the need for additional scanning.

Ibrahim and Bowman^7^ explored the potential of dual-energy CT for myocardial iron quantification using a gel-based phantom model. Their design employed a 0.5% agarose gel doped with 0.085 mM MnCl_2_, which was spiked with graded amounts (500-4500 mg) of ammonium iron (II) sulphate in 50 mL vials. While this approach provided an early proof-of-concept study, the physical properties of their gel-based model differed substantially from native myocardial tissue. In our study, we used homogenized myocardium from recently expired pigs to emulate realistic physiological imaging characteristics, potentially improving the external validity of our results.

Tsai et al.^8^ performed an *ex vivo* investigation using minced porcine myocardium enriched with iron. Authors observed strong linear correlations between attenuation and iron concentrations but did not investigate the potential of 3-material decomposition to elucidate potential presence of fat in the myocardium. Diffuse fat infiltration significantly lowers the CT attenuation of iron and is commonly encountered throughout the body including the myocardium, particularly in patients with obesity or metabolic disorders.^13^^,^^19^^,^^20^

In our study, iron concentrations varied between 0 and 20 mg Fe g^−1^ dry tissue, covering the physiological to pathological range reported for human myocardium.^3^^,^^12^ We incorporated a defined fat fraction of 5% into the myocardium representing a clinically realistic concentration^13^^,^^14^ to test the performance of 3-material decomposition under more difficult conditions. The ability to simultaneously characterize fat and iron content could prove valuable in patients with coexisting pathologies, such as metabolic syndromes and chronic anaemia caused by haemoglobinopathies, where both factors influence cardiac morphology and function.^20^ Moreover, for the cardiac fat component specifically, a recent review summarizes associations between epicardial and pericardial adipose tissue and cardiovascular outcomes, underscoring the need for standardized imaging and reporting to distinguish the 2 depots.^21^

CT-based quantification of myocardial iron should be considered as complementary to MRI-based myocardial tissue characterization rather than a potential replacement. T2*-MRI is likely to remain the reference standard for myocardial iron quantification, owing to the direct effect of iron on magnetization.^3^ However, access to this modality is limited, and cardiac MRI may be time-intensive, impractical or contraindicated in certain patients.^6^ In contrast, CT offers rapid data acquisition, wide accessibility, and compatibility with most cardiac devices—advantages that are particularly relevant in emergency care, intensive care, perioperative settings, or whenever prolonged MRI is impractical. Embedding quantitative iron mapping into routine cardiac CT examinations through coronary calcium scoring holds promise for opportunistic myocardial screening for iron and fat depositions, facilitating earlier detection in high-risk cohorts.

PCD-CT is particularly advantageous for myocardial characterization through the combination of high temporal resolution and inherent spectral data acquisition.^22–25^ In our study, a low-dose scan with an average CTDI_vol_ of 2.2 mGy was applied being in the typical range of calcium scoring protocol. As a next step towards clinical adoption, cross-calibration with T2*-MRI in patients, along with standardized quality control will be essential.^26^ In this context, any clinically applicable calibration must be established *in vivo*, as porcine myocardium does not fully replicate the biochemical composition and microstructural properties of human cardiac tissue, although the concentrations of fat and iron in our study were taken from previous work in humans. Accordingly, the calibration curves derived from our phantom provide a technical proof-of-concept but cannot be directly translated to patient imaging.

We must acknowledge the following study limitations. First, although efforts were made to replicate *in vivo* myocardial conditions as close as possible by using homogenized porcine myocardium and organic fat combined with iron (III) citrate, the model lacks the structural complexity, vascularization, and dynamic physiological properties of living cardiac tissue. Second, we have only used a single fat concentration to test the methods robustness. Third, despite careful mixing procedures, the phantoms exhibited some heterogeneity and macroscopic air bubbles attributable to the preparation process. To mitigate measurement inaccuracies due to these artefacts, various ROIs were placed in multiple slices around air bubbles ensuring representative sampling across probes. Fourth, potential oxidation or precipitation of iron which could alter iron distribution might have occurred prior to the scans. However, this was addressed by storing the phantoms at 2 °C and performing all scans within 24 h post-preparation. Fifth, we employed the only currently available dual-source PCD-CT for full clinical use from a single vendor, which limits generalizability to other CT systems. Sixth, the study lacked validation against established reference standards such as MRI-based iron quantification, which precludes direct comparison. Seventh, no test–retest measurements were performed, and thus intra-scanner repeatability could not be assessed.

In conclusion, our experimental study indicates that myocardial iron quantification by PCD-CT is feasible and yields accurate results across clinically relevant concentrations. Dual-energy-based, iron-specific 3-material decomposition eliminates the confounding effect of myocardial fat on iron quantification. While these findings are technically promising, translation to clinical practice will require validation in patients with myocardial iron overload, ideally in direct comparison with the current reference standard cardiac MRI.
